# Evidence that metallic proxies are unsuitable for assessing the mechanics of microwear formation and a new theory of the meaning of microwear

**DOI:** 10.1098/rsos.171699

**Published:** 2018-05-23

**Authors:** Adam van Casteren, Peter W. Lucas, David S. Strait, Shaji Michael, Nick Bierwisch, Norbert Schwarzer, Khaled J. Al-Fadhalah, Abdulwahab S. Almusallam, Lidia A. Thai, Sreeja Saji, Ali Shekeban, Michael V. Swain

**Affiliations:** 1 Max Planck Weizmann Center for Integrative Archeology and Anthropology, Max Planck Institute for Evolutionary Anthropology, Deutscher Platz 6, D-04103, Leipzig, Germany; 2 Smithsonian Tropical Research Institute, Luis Clement Ave., Bldg. 401 Tupper Balboa Ancon, Panama, Republic of Panama; 3 Department of Anthropology, Washington University in St Louis, Campus Box 1114, One Brookings Drive, St Louis, MO 63130, USA; 4 Department of Bioclinical Sciences, Faculty of Dentistry, Kuwait University, PO Box 24923, Safat 11310, Kuwait; 5 Saxonian Institute of Surface Mechanics SIO, Tankow 2, 18569 Ummanz, Rügen, Germany; 6 Department of Mechanical Engineering, College of Engineering and Petroleum, Kuwait University, PO Box 5969, Safat 13060, Kuwait; 7 Department of Chemical Engineering, College of Engineering and Petroleum, Kuwait University, PO Box 5969, Safat 13060, Kuwait; 8 Nanotechnology Research Facility, College of Engineering and Petroleum, Kuwait University, PO Box 5969, Safat 13060, Kuwait

**Keywords:** tooth wear, enamel, wear theory

## Abstract

Mammalian tooth wear research reveals contrasting patterns seemingly linked to diet: irregularly pitted enamel surfaces, possibly from consuming hard seeds, versus roughly aligned linearly grooved surfaces, associated with eating tough leaves. These patterns are important for assigning diet to fossils, including hominins. However, experiments establishing conditions necessary for such damage challenge this paradigm. Lucas *et al*. (Lucas *et al*. 2013 *J. R. Soc. Interface* **10**, 20120923. (doi:10.1098/rsif.2012.0923)) slid natural objects against enamel, concluding anything less hard than enamel would rub, not abrade, its surface (producing no immediate wear). This category includes all organic plant matter. Particles harder than enamel, with sufficiently angular surfaces, could abrade it immediately, prerequisites that silica/silicate particles alone possess. Xia *et al.* (Xia, Zheng, Huang, Tian, Chen, Zhou, Ungar, Qian. 2015 *Proc. Natl Acad. Sci. USA* **112**, 10 669–10 672. (doi:10.1073/pnas.1509491112)) countered with experiments using brass and aluminium balls. Their bulk hardness was lower than enamel, but the latter was abraded. We examined the ball exteriors to address this discrepancy. The aluminium was surfaced by a thin rough oxide layer harder than enamel. Brass surfaces were smoother, but work hardening during manufacture gave them comparable or higher hardness than enamel. We conclude that Xia *et al*.'s results are actually predicted by the mechanical model of Lucas *et al*. To explain wear patterns, we present a new model of textural formation, based on particle properties and presence/absence of silica(tes).

## 1. Introduction

Several decades of observational research on the surface textures of tooth enamel has resulted in the establishment of reliable techniques for three-dimensional description of damaged enamel surfaces [1]. The term ‘dental microwear’ is used to describe this general field, the prefix ‘micro-’ signifying that the events producing this wear are known to be microscopic [2]. The marks made on tooth enamel could be due to dietary components and extraneous grit or (bits of) teeth themselves [3,4]. Causation may remain contentious, but statistical correlations have been found between patterns of dental microwear and diet that are thought to be a function of food material properties. Specifically, the consumption of hard foods is associated with irregular, complex surface textures often marked by pits, while the consumption of compliant and tough foods is associated with anisotropic surface textures in which linear scratch-like features are aligned in roughly the same direction [1,5]. It has been hypothesized that differences in food material properties are linked to patterns of jaw movement, which in turn influence the nature of the resulting microwear [1,6,7]. As a result, dental microwear analysis has become a standard method of reconstructing diet in extinct vertebrates including fossil humans [8,9].

Recently, mechanical analyses have suggested that dental microwear (where ‘wear’ here signifies instantaneous loss of tissue) is controlled by the hardness and geometry of particles in the oral cavity, with the implication that particle mechanical properties may have a greater impact than food material properties on dental microwear textures [3,10,11]. If true, then the fundamental premise underlying the interpretation of dental microwear textures would be somewhat undermined, with the utility of microwear analysis as a means of reconstructing diet called into question. The mechanical analyses of Lucas and colleagues have themselves been challenged by tooth wear experiments involving contacts between enamel and metal balls [12]. Here, we continue this dialogue by evaluating the experimental evidence.

The mechanical argument of Lucas *et al*. [3] predicts that very small particles can abrade (i.e. wear) enamel at very low forces when those particles are sufficiently hard and angular. Importantly, ‘abrasion’ refers here specifically to the removal of enamel volume from the tooth crown, and does not include the marking of enamel without any immediate tissue loss, a phenomenon called ‘rubbing’ in the wear literature [13–15]. Specifically, an angular particle that is roughly twice as hard (or more) as enamel should detach chips of enamel from the crown, resulting in either irregular pits or, potentially, linear scratches with angular cross-sections. By contrast, particles lacking either sufficient hardness or angularity should not abrade enamel, but could produce rubbing marks with rounded contours. At one end of, and alongside such a groove, there is a ‘prow’ or ‘build-up’ of plastically deformed enamel that has been displaced by the advancing particle. Importantly, the hardness of enamel (range 3.5–5.5 GPa) far exceeds that of any plant tissue or plant product, which would include phytoliths based on published hardness data [3,10,16,17]. If so, no plant-based food would seem to be able to abrade enamel directly. Repeated rubbing could lead to wear at a slower, but currently unknown, rate. However, it is unclear how many further contacts might be required to dislodge elevated pieces of tissue. The issues of the quality of a contact, i.e. the mechanics involved, should be separated from the quantity of contacts. If a rubbing contact is far more frequent than an abrasive one, then the former could lead to more tissue loss. Experiments on this have been made [4], which will be discussed later.

In single contacts, predictions were validated experimentally by sliding individual phytoliths and grains of quartz dust across a polished enamel surface using a nanoscale mechanical tester. The phytoliths ranged in hardness from 0.43 to 4.24 GPa, while the hardness of the dust particles ranged from 10.1 to 14.1 GPa [3]. As predicted, the quartz particles, when possessing small, angular asperities, detached irregular chips of enamel, while the phytoliths produced rubbing marks on the tooth surface as the phytolith and enamel mutually deformed. The mechanics have subsequently been refined [11], and further experiments have shown that rounded quartz particles, as are common in nature, can easily fracture in the oral cavity producing angular shards that can then directly abrade teeth [10]. An implication of all of this work is that while phytoliths might be hard enough to produce linear scratch-like rubbing marks on tooth surfaces, neither they nor any plant tissues, including the densest and hardest seed shells [18,19], are hard enough to directly abrade enamel. Indeed, most plant tissues are too soft to even rub enamel, and thus their consumption may not leave behind any marks on teeth, or do so at a greatly reduced frequency. Insofar as the removal of enamel chips results in irregular scars on the tooth surface, it seems unlikely that plant tissues contribute much to the pitting often associated with complex surface texture. Thus, complex textures indicate the consumption more directly of grit rather than hard foods. Low complexity textures featuring linear marks could be caused by phytoliths or grit, but the former would produce rubbing marks while the latter would produce true abrasive scratches. These different types of marks could, in principle, be discriminated using nanoscale imaging, the sort provided by atomic force microscopy [3].

In contrast, more recent tooth wear experiments have purported to show that enamel can be directly abraded by sliding aluminium and brass balls, despite their measured hardness, 1.29 and 2.60 GPa respectively, being less than that of enamel and apparently being smooth-surfaced [12]. The aluminium spheres produced linear abrasive scratches that appear from published images to be approximately 1–2 µm in breadth, while the groove shown in the supplementary information of [12], produced by a brass sphere, was somewhat wider (less than 10 µm in breadth) with smooth profile and slightly raised sides, as in plastic rubbing. The results with aluminium, at least, challenge the above mechanical model on the stated grounds that enamel microstructure precludes enamel from deforming plastically, and that ‘Hydroxyapatite (HAP) crystallites are glued together by proteins, and tissue removal requires only that contact pressure be sufficient to break the bonds holding the enamel together.' Xia *et al*. conclude that ‘Dental microwear remains a valuable tool for reconstructing diets of fossils.'

What could explain this discrepancy from the theory? Several scale-related phenomena may be important. The hardness of some metals is much greater at the nanoscale due to an ‘indentation size effect' [20]. Also, the hardness of the spheres may not be uniform throughout their thickness. Moreover, the process used to manufacture the spheres may have resulted in work hardening. Additionally, it is possible that the surfaces of the spheres may have been oxidized. Aluminium oxide, in particular, is much harder than bulk aluminium [21–24] and spectra shown in electronic supplementary material, figure S1 of Xia *et al*. [12] clearly show oxygen atoms present on the aluminium surface. Lastly, a structure that is rounded on a macroscale (as the metal spheres) may not necessarily be smooth at the nanoscale. Surface asperities may be present of the appropriate size to produce abrasive wear.

This study tests the assumptions that the metal spheres used by [12] are completely smooth and lack surface asperities on a microscale, and secondly, that the surfaces of the spheres have the same hardness as their interior, and are thus truly insufficiently hard to produce abrasive wear on teeth. We test these assumptions using spectroscopy and nanoscale mechanical testing.

## 2. Material and methods

We obtained 1.8 mm diameter brass and 3.0 mm diameter aluminium spheres, essentially identical in composition to those used by [12], from the same manufacturer (Yue Li Hardware Products Co. Ltd, Yiwu City, Zhejiang Province, PR China). Indentation hardness was obtained by nanoindentation with a Hysitron Ubi700 (Minneapolis, MN, USA) with a Berkovich diamond tip, radius of tip curvature 150 nm, calibrated against fused quartz samples. The balls were indented orthogonal to their outer surfaces at extremely shallow depths, achieved by limiting forces to 250 µN for both aluminium and brass. To a depth *d* ≤ 50 nm, the contact was analysed as a contact between the spherical tip of the indenter and the flat surface of the oxide film. This is a commonly adopted approach [25] to the conditions for such shallow contacts, where the radius of contact *a* in the plane of the oxide surface can be approximated as *a *= (*Rd*)^0.5^, where *R* is the radius of the indenter tip. The area of contact is $\pi a^{2}$, thus giving an expression for the calculated average pressure of contact as F/πdR, where *F* is the force. A scanning electron microscope (SEM) was used to examine the outer surfaces of the balls in regions that were indented, along with energy-dispersive X-ray spectroscopy (EDS, Oxford Instruments, Abingdon, UK) to ascertain the elemental composition of these surfaces.

## 3. Results

**Figure 1.**
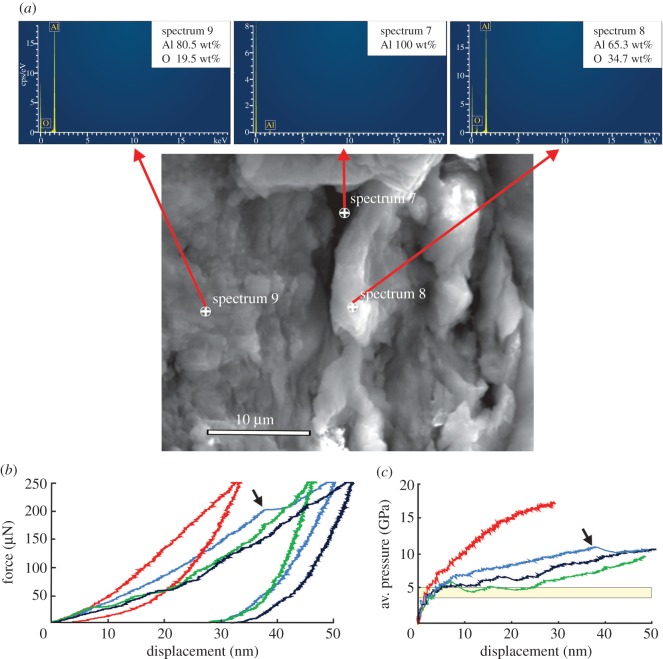
Detection of the surface oxide film on aluminium balls and its properties. (*a*) The oxide layer is thin and incomplete, as shown by EDS. The location at spectrum 7 shows apparently uncoated aluminium, while those at spectra 8 and 9 show the rough nature of the oxide coat. (*b*) Four nanoindentation force–displacement curves on the outer surface of an aluminium ball during loading and unloading. Three of the curves show force declines associated with pop-in cracks, the most obvious shown by an arrow. (*c*) Replots the vertical axis for the loading part of the curves in (*b*) in terms of the average contact pressure. This exceeds the hardness range for enamel (yellow rectangle) after just a few nanometres of indentation.

SEM–EDS analysis showed that the aluminium balls were coated with a very thin film (figure 1*a*). Surface asperities were evident on the images, which EDS analysis indicated to be an oxide layer (figure 1*a*). A few of the probes did not reveal oxygen, only aluminium, indicating that this film was both shallow and incomplete (figure 1*a*). During indentation, the displacement often displayed sharp steps indicating pop-in events typical of cracks in a brittle film (arrow on the blue curve in figure 1*b*, and force dips and plateaus on the blue, green and black curves in figure 1*c*). Despite this, average contact pressures typically rose above 5 GPa (figure 1*c*), continuing to rise when no cracking was seen (red curve in figure 1*b,c*) to levels greater than 10 GPa.

In contrast, the surface layer of the brass balls was smooth. Monotypic indentations (*n* = 43) revealed a reduced elastic modulus typical of brass: mean 111.9 (s.d. 16.1) GPa, yet with a mean hardness of 4.53 (s.d. 1.1) GPa with a range of 2.9–7.5 GPa, which overlaps or exceeds that of enamel. The force–displacement curves in brass did not show any pop-in events associated with cracking of any surface layer, but only a transition from elastic to plastic deformation, thus providing smoother conditions of contact.

## 4. Discussion

Aluminium and brass have long been employed as standards for assessing the hardness of dental tissues [26]. However, when used to assess wear resistance, the bulk hardness of these metals, which is what is usually measured, does not necessarily represent the surface properties of objects made from them. The critical aspect of any aluminium surface is the oxide layer that will grow immediately after the metal is exposed to air. Unlike the pure metal, the oxide layer is brittle, as shown by the pop-in events associated with cracking in figure 1*b,c*. Aluminium oxide coatings are formed in a number of metastable structural types [21], of which the γ form is most likely to result from low-temperature manufacture. Such films can have a nanohardness anywhere in the range 4–25 GPa, tending towards the upper limit when fully dense and crystalline and towards the lower bound when amorphous and highly porous [21–24]. After just a few nanometres of penetration, the lower end of the pressure range exceeded the hardness of enamel (figure 1*c*), suggesting that even a highly porous oxide film would abrade enamel, in accordance with [3]. However, the porosity, micrometre size thickness and brittleness of the oxide film in our sample (figure 1*a*) confounded accurate hardness measurement. While the contact pressure exceeded 10 GPa by 10 nm depth, there was minimal indication of plastic deformation prior to a pop-in event. Thus, the hardness of the film could not be determined with certainty because hardness is defined as resistance to plastic indentation and deformation prior to pop-in was largely elastic.

The approximation of the rounded Berkovich indenter tip to a partial sphere is very accurate at shallow depths. Attempts to define the shapes of worn tips more precisely have produced contrasting results: one group [27] found that a spherical approximation slightly underestimated the subsurface resolved shear stresses while the other [28] states that it produces a slight overestimate. Clearly, each tip might be unique in form. However, neither paper [27,28] deals with the issue of contact pressure versus depth, which is our focus. Further, neither had to consider an oxide film [29]. Such a film increases the load needed to initiate dislocation motion and pop-in associated with oxide film fracture [30]. Problems, particularly with the issue of residual stresses in these films, remain unresolved.

**Figure 2.**
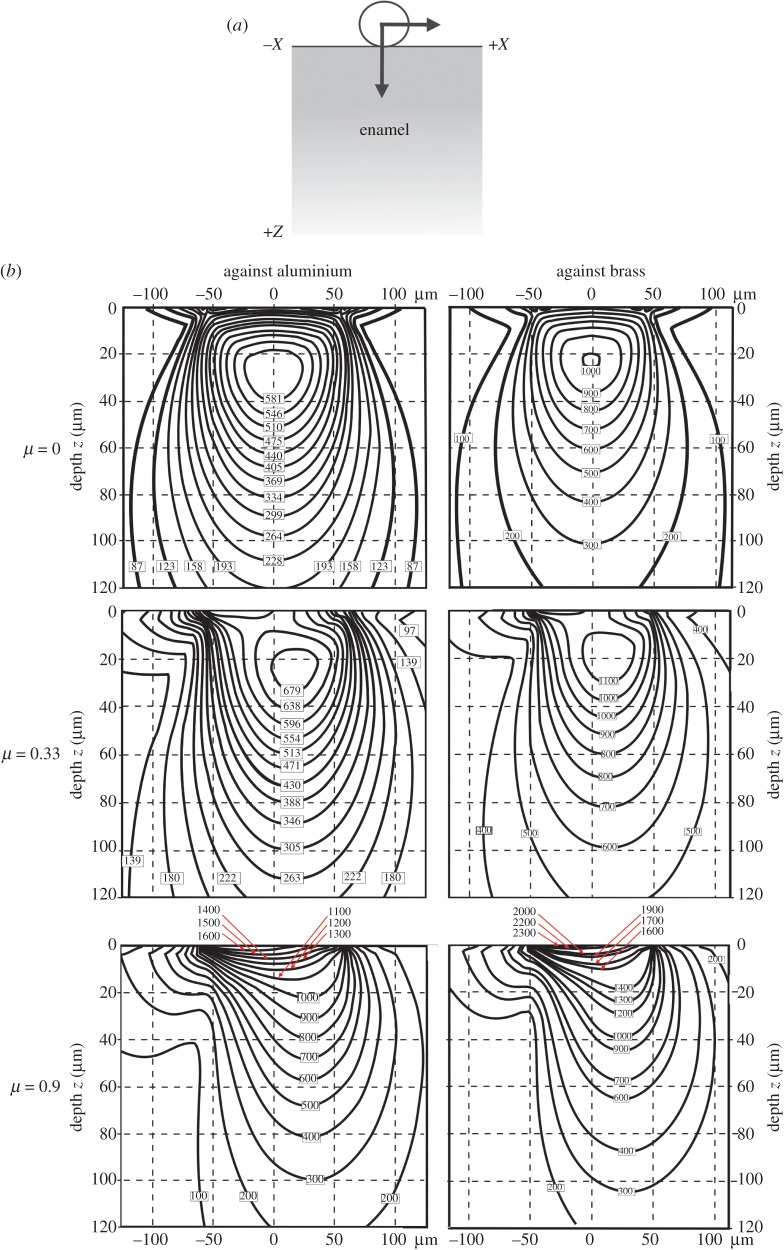
Stresses induced in enamel from contact with an uncoated aluminium and brass ball. (*a*) A metal ball is subjected to a normal force on enamel, but also to a variable lateral force, so as to produce different values of the coefficient of friction *µ*. (b) von Mises stress maps in MPa, calculated from [31], assume quasi-static conditions and the following material properties: *enamel:* elastic modulus *E* = 70 GPa, Poisson's ratio *ν *= 0.25*; aluminium ball: E* = 70 GPa, *v* = 0.33, radius *R* = 1.5 mm; *brass ball*: *E* = 120 GPa, *v* = 0.33, *R* = 0.9 mm. For all cases, normal force = 7 N (as [12]), the lateral force being adjusted to change *µ*. For any given value of *µ*, stresses in enamel are higher for contacts with brass. When *µ* reaches 0.9, they exceed the yield stress of enamel (1400 MPa).

**Figure 3.**
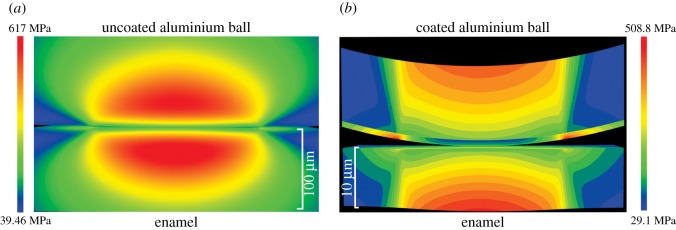
Visual impression of the stresses produced in both an aluminium ball and enamel (*a*) without an oxide film and (*b*) with a 1-µm oxide film coating the ball evenly. Note that calculations in (b) are only taken to 10 µm depth in order to visualize the film. At greater depths, stresses in the enamel from the contact in (b) also rise to 619 MPa.

**Figure 4.**
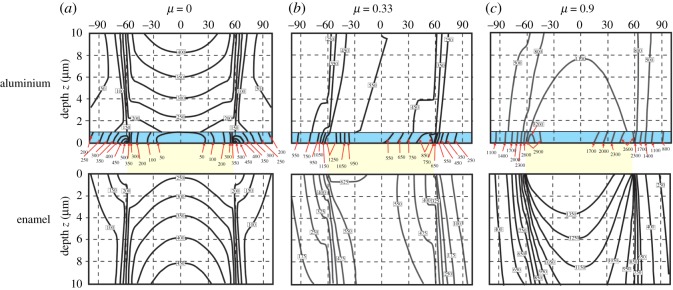
Maps of von Mises shear stresses (in MPa) developed within both a coated aluminium ball and enamel. Properties and loading conditions are as in figure 2, with the additional 1 µm thick oxide coating (shown in blue) having *E* = 200 GPa and *ν* = 0.2. Stresses are only mapped up to 10 µm deep to the surface. The aluminium ball (upper plots) is shown inverted over the enamel surface. Contact radii are 59 µm in all cases (indicated by yellow shading). (*a*) Pure vertical loads. Maximum shear stresses are 619 MPa in the aluminium ball, but additional secondary stresses peak at 557 MPa within the oxide film at the edge of the radius of contact. (*b*) Coefficient of friction of 0.33 results in a non-symmetric stress pattern where the maximum shear stresses are now 680 MPa in the ball, well below the thin stiffer layer. However, additional much higher secondary maximum stresses of 1540 and 890 MPa develop within the oxide film at the edges of the radius of contact. (*c*) Coefficient of friction of 0.9 results in a more asymmetric stress field with the maximum shear stresses in the ball having a maximum just below the oxide film of 1520 MPa, while in the film itself, they have increased to 3410 and 2840 MPa at the edges of the radius of contact.

Further contact details are relevant to the results of [12]. The contact pressure of approximately 0.98 GPa in their experiments exceeds that which will lead to the onset of plastic deformation of the aluminium. This is because yield of a metal during spherical contact typically initiates subsurface, at approximately half the radius of contact, when contact pressures exceed 0.4 of the metal hardness. A thin micron oxide layer on the aluminium ball has very minimal influence on the subsurface contact stresses in the vicinity of the maximum shear stresses as the contact radius is much greater than the film thickness. Such subsurface plastic deformation, particularly at high coefficients of friction, would enhance the break-up of the oxide film, exposing sharp flakes of oxide to the enamel and leading to abrasion. Unfortunately, in the sliding experiments of aluminium and brass balls across the enamel surface [12], the coefficient of friction associated with sliding was not determined. This has a profound influence on the magnitude and location of the subsurface shear stresses in the opponent substrate [31], in this case enamel. To support this, we generated von Mises stress maps in enamel for the sliding of both aluminium and brass balls against it during zero, low and high friction contacts. These stress maps are based on analytic functions, not finite-element modelling [32], and visualized with specialized software (www.siomec.de/filmdoctor). Figure 2 shows results for both metals, but without considering an oxide coating on the aluminium. Stresses due to both metals rise with the coefficient of friction *µ* (figure 2*b*), with stresses above 1.4 GPa certain to lead to plastic disruption of the enamel structure [33]. In sliding contacts, aluminium generally develops very high coefficients of friction (approaching 1.0), whereas brass values are much lower (approx. 0.3). Low values of the coefficient of friction, as presumed for brass, have only a minor effect on the magnitude and location of the shear stresses, whereas higher values associated with aluminium greatly increase their magnitude and move them much closer to the surface (figure 2*b* bottom left). To indicate how the latter would contribute significantly to the break-up of the oxide film, we show its effect both visually in terms of the whole contact (figure 3) as well as in quantitative detail in figure 4. Plots of the stresses within the thin brittle oxide film as a function of the coefficient of friction *µ* (figure 4) show that their magnitudes within the oxide film are greatest at the edge of contact and become more non-symmetric. At the highest value of *µ*, the stresses at the trailing edge of contact within the film are more than six times greater (3400 MPa) than in the absence of friction (540 MPa).

Oxide layers that grow on brass are much smoother and softer than those on aluminium. Instead, the critical aspect for brass neglected in [12] is work-hardening. The bulk hardness of α-brass can more than triple after being worked and this is known to be important for the cutting process [34]. In addition, the hardness of both metals can be higher at the nanoscale which is important in understanding their ability to cut into enamel or not [35]. At any given coefficient of friction, stresses in enamel are higher for brass than aluminium contacts (figure 2*b*).

We conclude that the sharp edges in the outer brittle aluminium oxide, both as received and also after the asperities and brittle oxide flakes break off during sliding, would result in abrasive conditions suitable to produce the wear marks observed by [12]. Work-hardened brass is consistent with results shown in the supplementary information of [12], in which the troughs shown appear to be at least in part the product of rubbing, evinced by prowing [3] at the edge of grooves and the troughs' comparatively shallow and wide profile. No direct evidence of abrasion is described for the brass experiments [12], but given that the brass sliding experiments were repeated 60 times on the same enamel surface, abrasion due to fatigue is not unexpected. Such repetition confounds the ability of those experiments [12] to characterize the very phenomena they sought to observe. Furthermore, at the level of the quality of the contact between brass or aluminium and enamel, our results clearly allow rejection of the assumptions that the metal spheres used in the experiments of [12] lack either surface asperities or elevated surface hardness. These assumptions underlie their interpretations of their experimental results, and thus the present study renders their interpretation invalid. Indeed, the mechanical model of [3] actually predicts the type of results obtained by [12] in their metal sphere experiments.

The sliding experiments on metallic balls [12] were accompanied by similar experiments using silicon dioxide spheres [12]. The hardness of these balls is reported [12] to be higher than enamel (5.7 GPa), although no direct measurements of hardness or modulus were made on the spheres and stated values were estimated from experiments performed on deposited silicon oxide films [36]. The spheres presumably lack asperities, but were only 1–2 µm in diameter, below the range of wind-blown dust. During sliding, the spheres damaged individual enamel crystals on a submicrometre scale (range approximately 10–100 nm), which is orders of magnitude smaller than features observed in dental microwear analysis. Nothing in the mechanical model of [3], which describes wear phenomena at the micro- rather than nanoscale, precludes the type of damage observed by Xia *et al.* [12] in their experiments with silicon dioxide spheres. Indeed, whereas the polished enamel surfaces in their experiments are flat on a microscale, they are not obviously flat on a nanoscale (e.g. their figure 4), and it may be that asperities in the enamel surface (rather than the silicon dioxide ball) contribute critically to the wear observed. This is certainly a phenomenon worth studying, but it does not relate obviously to the surface textures that were typically classified as microwear. Ultimately, the key drawback of [12] is simply the use of inaccurate proxies for the actual candidate particles responsible for tooth wear. Employing actual phytoliths [3] seems to produce results that conform with theoretical predictions at the scale of wear marks.

Thus, none of the results of [12] contradict the finding of [3]. However, this does leave the matter of whether a rigid-plastic analysis [14] is germane to tooth wear [3]. Any material, whether metal or a hierarchical mineralized composite like enamel [37] will behave inelastically at a fine-enough scale [38]. The latter shows that for smaller contact dimensions, the stress for the onset of inelastic response rises, as also does the extent of shear strain deformation. The wear model presented in [12] was based at the first hierarchical level, assuming that HAP crystals can be removed easily by overcoming the protein bonds that hold the crystals together. This is either a simplification or misreading of the role of the protein matrix around HAP crystals: biological mineralized tissues do not resemble artificial composites where nanoparticles are embedded in ‘glue' because they would not otherwise have different properties at different scales [39]. Inter-crystalline proteins in biocomposites non-homogeneously fill the free space between crystals and are capable of rearranging and temporarily sacrificing bonds to toughen enamel. Added to this, HAP crystals are arranged imperfectly; interlocking unusual shapes, lateral branches and bifurcations all lead to crystals holding together to form a cohesive structure. We are not naive, and understand that contacts inducing elastic movements in enamel may fracture or dislodge individual or at times small clumps of crystals, but these events will be limited to very small enamel particles [12]. This is nothing compared to that seen with contacts with harder particles like grit or dust and they are unlikely to contribute significantly to the micro-striations or complex textures associated with microwear investigations.

Xia *et al*. [12] point to ‘smoking gun' evidence of phytoliths abrading enamel in that they have been found embedded in enamel surfaces, with marks trailing behind them [40]. However, there is no way of determining from the scale of these images whether the linear marks are true abrasive scratches or are rubbing marks. More to the point, embedding is circumstantial evidence that the phytolith and enamel surface are mutually plastically deforming with the resulting conformity of shape contributing to the phytolith–enamel adhesion.

Naturally, the mechanical model of [3] requires further testing in order to assess its broader biological applicability, particularly insofar as the model challenges the conventional wisdom concerning the use of dental microwear texture analysis as a tool to reconstruct diet in extinct vertebrates, including fossil hominins. To this end, several recent studies are very relevant [4,41–44].

The wear effect of phytoliths, has been simulated by a mechanical chewing machine that replicated the movements of horse jaws, producing a year's worth of chews over a relatively short period [4]. Experiments simulated five different types of ‘diet,' namely, a low phytolith diet (alfalfa), a high phytolith diet (grass), a higher phytolith diet (grass plus rice husks), a high phytolith diet plus sand and a pure tooth-on-tooth regime (termed ‘attrition') in which no plants or grit were processed (meaning that the only source of wear was direct tooth-on-tooth contact). Several important micro- and macrowear patterns were demonstrated. Regarding microwear, the presence of sand, and pure attrition, produced wear textures with a greater number of large or very large pits, as well as shorter scratch lengths, than most chewing simulations in which plant tissues were processed in the absence of sand. Moreover, the processing of plant tissues produced textures with more scratches than those without any plant tissues, and, in the absence of sand, none of the plant tissue diets differed significantly from each other in any of the measured microwear variables. Thus, in these experiments, microwear variables were influenced primarily by the presence/absence of phytoliths (even in low density) and sand. Regarding macrowear, it has been estimated that pure tooth contact wore enamel at a base rate of 0.58 mm per year, whereas a ‘low' phytolith diet wore it at only a third of this, indicating that the plant tissue buffered teeth against wearing each other [4]. However, ‘high' and ‘very high' phytolith diets wore the teeth at a rate of roughly 7–8 times the rate of attrition alone, while the addition of sand wore the teeth at more than 15 times. These data suggest that a high phytolith diet does eventually lead to tooth wear, although it is not clear from these data whether this is due to direct abrasion [12] or a combination of rubbing plus fatigue [3]. Regardless, it is clear that sand wears enamel at a much faster rate than phytoliths (twice as fast, in this experiment), which is compatible with the predictions of [3]. Interestingly, macrowear patterns did not correspond to microwear patterns. For example, the low and high phytolith diets had statistically indistinguishable microwear patterns, but radically different macrowear patterns, with the same true for pure attrition versus the sand diet.

Hua *et al*. [41] added grit to foods, then simulated human mastication in a chewing machine that allowed for control of the angles of approach during cycles. They compared microwear textures both before and after 100 simulated chews, finding that perpendicular approach angles generated pits, parallel angles generated scratches with oblique angulations generally creating a mixture of both. This was interpreted to indicate that different food types and abrasiveness are likely to influence the formation of microwear. This interpretation relies on the assumption that tough foods are processed with transverse jaw movements while the consumption of hard foods is associated with more vertical jaw movements [6,7]. However, experimental studies of both humans [45] and non-human primates [46] have found the opposite: harder foods are chewed with more lateral movement. Occlusal relief may influence microwear as, to some extent, teeth act as guides during chewing [47]. By virtue of their shape, low cusped bunodont teeth may offer greater degrees of freedom for food and tooth movement during mastication when compared to high crested teeth. This relationship in primates should be maintained throughout life because, even as teeth wear, they tend to maintain general occlusal slope patterns [47]. If this holds true, then some portion of microwear patterning may reflect dental morphology rather than food material properties *per se*. This indicates that current predictions about the causes of microwear textures may be somewhat flawed.

It is known from a wear model with a strong theoretical basis that, though the hardness of the wearing agent is a key factor, the angularity of contacts and elastic modulus mismatch matter too [42]. The idea that foods of low modulus alone, without consideration of anything else, could induce microwear has been investigated experimentally [43] by taking large food items of varying stiffness and compressing them to failure against enamel 10 times in succession. Observations of surface textures before and after testing revealed the formation of some microwear patterns although the complexity of such patterns was not obviously related to food item stiffness. Although it has been inferred that such foods are capable of indenting and damaging enamel [47], damage in these experiments is orders of magnitude smaller than food items that were compressed to fracture and it is unclear what might be causing such markings, especially as the authors could not completely control for external silicates and made no attempt to measure the presence of internal hard particles, such as phytoliths in seed casings [19].

Merceron *et al*. [44] used long-term sheep feeding studies to assess if dust particulates can interfere with the dietary signal conveyed by surface textures. They added fine scale grit and dust (less than 100 µm in diameter) to both browse (red clover) and grazing (grass) fodder to see if it altered the microwear signal from dust-free vegetation. The amounts of dust were derived from studies of the harmattan wind deposits in order to replicate natural conditions. Although the addition of dust did alter some measures of surface texture, none of these were shown to be significant, prompting the authors to declare that ‘dust does not matter' and that dietary signals (meaning the amount of phytoliths present) prevail in the presence of external abrasives. There are some other interesting results in the study, however. First, anisotropy appears to be inversely proportional to dietary toughness, which is the opposite of what is predicted by the conventional wisdom of dental microwear analysis [6,7]. Second, surface complexity appears to be inversely proportional to phytolith concentration, while anisotropy is directly proportional to it. These findings are compatible with the predictions of [3]. Comparing this result to [4], it is conceivable that in this particular experiment, the number of phytoliths in the diet simply overwhelmed the number of particles of quartz dust added. Lastly, the addition of dust appears to be associated with a slight reduction in complexity and a slight increase in anisotropy. It is not obvious why this is true, but perhaps these results reflect the fact that the dust particles used in the experiments were fine grained. More research is needed on how particle size influences the mechanics of microwear formation.

Resolution of the mechanics underlying dental microwear formation has particular salience to our understanding of human evolution. Dental microwear analysis has featured prominently in reconstructions of early hominin diets and dietary adaptations, but these analyses seem to contradict interpretations from functional anatomy. In particular, most australopiths lack complex dental microwear textures (which are conventionally associated with hard object feeding). Instead, their textures somewhat resemble those seen in primates that eat compliant and tough foods like leaves [5,48,49]. These data, in combination with stable carbon isotope analyses indicating that some species consumed a very high proportion of C4 foods (either plants using the C4 photosynthetic pathway or the animals that ate those plants), has led to the hypothesis that many australopiths incorporated compliant and tough grass tissues into their diet [6], and that this in turn influenced morphological adaptations in some early hominin species. In contrast, analyses of occlusal morphology suggest that the blunt teeth of australopiths (especially those in the genus *Paranthropus*) are not well suited for fracturing leafy vegetation [50–52], while studies of feeding biomechanics show that australopith crania are well suited for generating and resisting high bite forces [19,53–56]. These lines of evidence together suggest that australopiths are adapted to consume hard foods, with grass seeds being a potential source of the C4 isotopic signal seen in many species [19]. Recently, however, Ungar & Hlusko [57] have proposed that the reduction of shearing crests in the putative *Australopithecus afarensis* to *Paranthropus boisei* lineage is a developmental consequence of evolving thick enamel to resist abrasion, and that a large occlusal surface would, in part, compensate mechanically for the loss of dental topography that would facilitate the processing of tough, fibrous vegetation. As noted by [57], there is no comparative evidence that would support this hypothesis, but their hypothesis poses a testable mechanical prediction, namely, that large, flat teeth are more efficient than small flat teeth at processing leafy vegetation (although still less efficient than teeth with shearing crests). According to them, a morphology that is suboptimal for processing tough, compliant food would nonetheless provide a selective advantage by prolonging tooth life. Left unstated is the fact that complex microwear textures (presumably indicating a hard food diet, according to conventional wisdom) are found in *P. robustus,* a species that is anatomically and functionally very similar to *P. boisei.* If these two species are close relatives, as most phylogenetic analyses suggest [58–61], then this hypothesis implies that *Paranthropus* evolved a morphology that was poorly designed for processing the compliant, tough foods in its diet, but that then was co-opted to process a hard food diet for which it is much better configured (meaning that the evolution of morphology that is optimally suited to the diet of a given species cannot be explained by that diet). Alternatively, if the two *Paranthropus* species are not closely related to each other, then they will have converged on a very similar feeding morphology despite having very different diets. Neither scenario is especially compelling. Ultimately, the hypothesis of Ungar & Hlusko [57] rests critically on an interpretation of dental microwear in which hard foods were not an important component of the diets of *P. boisei* and its ancestors. If, however, the absence of complex microwear is compatible with hard foods in the diet, then the premise of Ungar & Hlusko's [57] hypothesis disappears. Thus, an enhanced understanding of the mechanics of microwear formation will contribute significantly to our understanding of human evolution.

In sum, the results of [12] do not pertain to the question of whether or not dental microwear textures provide dietary information. The fault may lie in conventional interpretations of microwear, which assume that differences in microwear texture are caused by jaw kinematics that respond to varying food material properties. As stated above, the current viewpoint [7] has it that tougher foods are chewed with wider jaw excursions, but this is contradicted by experiment in humans and capuchin monkeys that demonstrate the reverse pattern [45,46].

**Figure 5.**
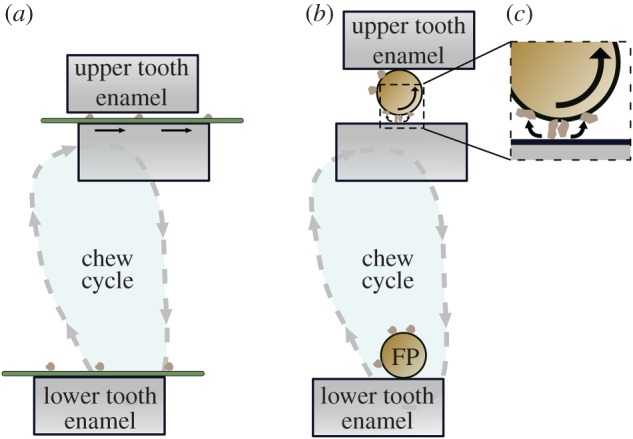
Simple schema to show the effect of food particle (FP) shape on rolling versus sliding wear. (*a*) A thin sheet such as a leaf (green) with randomly located adherent grit is raised on a lower tooth towards the upper at an angle. The normal load embeds the grit while the lateral force causes leaf and grit to slide together, so producing grooves or scratches. (*b*) A small food particle of isodiametric shape (brown), again coated with grit, is carried similarly towards an upper opposing tooth. The jaw movement trajectory encourages the seed to roll. (*c*) The embedded grit fractures at low loads; the resulting sharp fragments produce abraded pits.

With the above in mind, we present a new hypothesis as to how diet affects patterns of microwear features on teeth, based on plant food particle shapes that permit or obstruct the rolling of these particles. We propose that the key variable affecting the formation of dental microwear textures is the regularity or irregularity of loading during contacts between tooth surfaces and either grit particles or phytoliths. That regularity, or lack thereof, is in turn primarily a function of food shape. For example, when food takes the form of a sheet, such as a leaf or a grass blade, then grit that is embedded in the food, and/or phytoliths present within its outer structure, contact the teeth primarily when opposing tooth surfaces are in close proximity and sliding past each other (figure 5*a*). Note that this sliding is not necessarily related to pronounced transverse jaw movements; high crowned teeth with steep occlusal surfaces might not allow such movements, yet opposing facets would certainly be moving nearly parallel to each other. This type of contact should produce regular loading conditions that promote the formation of either abrasive scratches or linear rubbing marks (grooves) aligned in the same direction. Such features would contribute to highly anisotropic microwear textures. If phytoliths cause this damage, then these grooves would tend to be regular in density because phytoliths are regularly distributed in the leaf, whereas grit is randomly located and can cause scratches. In contrast, isodiametric (i.e. roughly spherical or polyhedral) food objects may roll between the teeth (with associated large lateral jaw excursions) such that they, or their fragments, break at their weakest positions [45]. Such rolling, even if a partial rotation, should produce unpredictable contacts between grit particles, phytoliths and teeth (figure 5*b,c*). For objects like large seeds, very high forces, into the high hundreds of newtons, can be produced in such circumstances [62]. Imagine grit or dust adhering to these seeds at random surface locations. These can easily embed in the woody coverings of seeds because these are much less hard than enamel. As the seeds roll, these grit particles fracture at sub-newton loads [10], so generating sharp edges that can produce abrasive pits (by mechanisms given by [63]) as the food particle rolls across the enamel (figure 5*c*). Phytoliths (when present, always close to the surface of seeds [19]) could also indent the enamel to produce blunt depressions. However, these would tend to lack fractures and, being of consistent density within any given seed, might not lead to the irregular distributions often seen in pitted wear. Further, small pitted indents like this that lack fractures, produced in experiments, tend to heal without damage to the enamel, so conceivably removing evidence of their formation [64]. Nothing particular ties this explanation to seeds *per se*; it could as well relate to any foods of such shape, including small fruits [50] because they will also tend to roll, not slide. Moreover, as an isodiametric food object (or any food object with substantial thickness relative to its length) is compressed between opposing tooth surfaces, lateral displacements of the food tissue might likewise produce irregular contacts between grit/phytoliths and tooth surfaces. Note that food hardness is not central to this model of microwear formation. Lateral displacements of food tissues are expected in soft foods (i.e. a bite-sized fragment of a fruit), meaning that so long as abrasive particles are present in the oral cavity, tooth wear could ensue. Minimally, therefore, the consumption of isodiametric (or otherwise thick) food objects should not be associated with highly anisotropic dental microwear textures. Whether or not the complexity of those textures will be high may depend on other variables, such as the relative proportion of phytoliths to grit in the oral cavity, and perhaps also the size of the grit particles (large grit particles might themselves roll, creating irregular contacts).

Our hypothesis allows for the possibility that dental microwear textures preserve dietary information, namely, that highly anisotropic textures probably indicate the consumption of film-like tissues (leaves, grass blades, etc.). Yet, our model differs fundamentally from conventional explanations of microwear textures because variables like food toughness and hardness do not play a role. The presence or absence of texture complexity seems unlikely to be informative about the presence or absence of hard foods in the diet.

## Data accessibility

Our data are deposited at Dryad: http://dx.doi.org/10.5061/dryad.72431 [65].
